# Committee on Organization of the Ninth International Medical Congress, to Be Held in Washington, D. C., in 1887

**Published:** 1885-01

**Authors:** J. S. Billings

**Affiliations:** Secretary-General; Washington, D. C.


COMMITTEE ON ORGANIZATION OF THE NINTH INTERNATIONAL MEDICAL CONGRESS, TO BE HELD IN WASHINGTON, D. C., IN 1887. *Preliminary Notice.*

The Committee on organization of the Ninth International Medical Congress, to be held in the United States in 1887, met in Washington, D. C., on November 29, 1884, for the determination of the general plan of the Congress, the election of Officers of the Committee, who will be nominated to fill the same offices in the Congress, and the consideration of questions of finance.

The following rules were adopted:

1. The Congress will be composed of members of the regular medical profession who shall have inscribed their names on the Register of the Congress, and shall have taken out their tickets of admission. As regards foreign members, the above conditions are the only ones which it seems, at present, expedient to impose.

The American members of the Congress shall be appointed by the American Medical Association, by regularly organized State and local medical societies, and also by such general organizations relating to special departments and purposes as the American Academy of Medicine, the American Surgical Association, the American Gynæcological, Ophthalmological, Otological, Laryngological, Neurological, and Dermatological Societies, and the American Public Health Association; each of the foregoing Societies being entitled to appoint one delegate for every ten of their membership.

The members of all special and subordinate committees, appointed by the General Committee, shall also be entitled to membership in the Congress, together with such other persons as may be specially designated by the Executive Committee.

All Societies entitled to representation are requested to elect their delegates at their last regular meeting preceding the meeting of the Congress, and to furnish the Secretary-General with a certified list of the delegates so appointed.

2. The work of the Congress is divided into eighteen sections, as follows, viz.:

1. Medical Education, Legislation and Registration, including methods of teaching and buildings, apparatus, etc., connected therewith.

2. Anatomy.
3. Physiology.
4. Pathology.
5. Medicine.
6. Surgery.
7. Obstetrics.
8. Gynæcology.
9. Ophthalmology.
10. Otology.
11. Dermatology and Syphilis.
12. Nervous diseases and Psychiatry.
13. Laryngology.
14. Public and International Hygiene.
15. Collective Investigation, Nomenclature, and Vital Statistics.
16. Military and Naval Surgery and Medicine.
17. Experimental Therapeutics and Pharmacology.
18. Diseases of Children.

3. The general meetings will be reserved for the transaction of the general business of the Congress and for addresses or communications of scientific interest more general than those given in the sections.

4. Questions which have been agreed upon for discussion in the sections shall be introduced by members previously nominated by officers of the section. The members who open discussions shall present a statement of the conclusions which they have formed as a basis for debate.

5. Notices of papers to be read in any one of the sections, together with abstracts of the same, must be sent to the Secretary of that section before April 30, 1887. These abstracts will be regarded as strictly confidential communications, and will not be published until the meeting of the Congress. Papers relating to questions not included in the list of subjects suggested by the officers of the various sections will be received. Any member, after April 30, wishing to bring forward a subject not upon the programme, must give notice of his intention to the Secretary-General at least twenty-one days before the opening of the Congress. The officers of each section shall decide as to the acceptance of any communication offered to their section, and shall fix the time of its presentation. No communication will be received which has been already published, or read before a Society.

6. All addresses and papers, read either at general meetings or in the sections, are to be immediately handed to the Secretaries. The Executive Committee, after the conclusion of the Congress, shall proceed with the publication of the transactions, and shall have full power to decide which papers shall be published, and whether in whole or in part.

7. The official languages are English, French, and German.

No speaker shall be allowed more than ten minutes, with the exception of readers of papers and those who introduce debates, who may occupy twenty minutes.

8. The rules, programmes, and abstracts of papers will be published in English, French, and German.

Each paper or address will appear in the Transactions in the language in which it was delivered by the author. The debates will be printed in English.

9. The officers of the General Committee on Organization are a President, three (3) Vice-Presidents, a Secretary-General, and a Treasurer, and those elected to these positions will be nominated by the General Committee to hold the same offices in the Congress. All vacancies in these offices shall be filled by election.

10. There shall be an Executive Committee, to be composed of the President, Secretary-General, and Treasurer of the General Committee, and of four other members, to be elected by the General Committee. The duties of the Executive Committee shall be to carry out the directions of the General Committee; to authorize such expenditures as may be necessary, and to act for the General Committee during the intervals of its sessions, reporting such action at the next meeting of the General Committee.

11. There shall be a Standing Committee on Finance, composed of five members, to be appointed by the President, subject to the approval of the Executive Committee.

12. Those who are elected as Chairmen of the several sections shall be thereby constituted members of the General Committee.

The officers elected are as follows:

*President.*—Dr. Austin Flint, Sr., of New York.

*Vice-Presidents.*—Dr. Alfred Stillé, of Philadelphia; Dr. Henry I. Bowditch, of Boston; Dr. R. P. Howard, of Montreal, Canada.

*Secretary-General.*—Dr. J. S. Billings, U. S. Army.

*Treasurer.*—Dr. J. M. Browne, U. S. Navy.

*Members of the Executive Committee,* (in addition to the President, Secretary-General, and Treasurer.)—Dr. I. Minis Hays, of Philadelphia; Dr. A. Jacobi, of New York; Dr. Christopher Johnston, of Baltimore; Dr. S. C. Busey, of Washington.

The Executive Committee will proceed at once to complete the work of organization.

J. S. BILLINGS,
*Secretary-General.*

WASHINGTON, D. C., Dec. 1, 1884.

